# Neuroimaging studies of the striatum in cognition Part I: healthy individuals

**DOI:** 10.3389/fnsys.2015.00140

**Published:** 2015-10-08

**Authors:** Jean-Sebastien Provost, Alexandru Hanganu, Oury Monchi

**Affiliations:** ^1^Department of Psychology, Faculty of Arts and Sciences, University of MontrealMontreal, QC, Canada; ^2^Centre de Recherche de l'Institut Universitaire de Gériatrie de Montreal, Université de MontrealMontreal, QC, Canada; ^3^Department of Clinical Neurosciences, Department of Radiology, Cumming School of Medicine, University of CalgaryCalgary, AB, Canada; ^4^Hotchkiss Brain Institute, University of CalgaryCalgary, AB, Canada

**Keywords:** caudate nucleus, putamen, prefrontal cortex, striatum, neuroimaging

## Abstract

The striatum has traditionally mainly been associated with playing a key role in the modulation of motor functions. Indeed, lesion studies in animals and studies of some neurological conditions in humans have brought further evidence to this idea. However, better methods of investigation have raised concerns about this notion, and it was proposed that the striatum could also be involved in different types of functions including cognitive ones. Although the notion was originally a matter of debate, it is now well-accepted that the caudate nucleus contributes to cognition, while the putamen could be involved in motor functions, and to some extent in cognitive functions as well. With the arrival of modern neuroimaging techniques in the early 1990, knowledge supporting the cognitive aspect of the striatum has greatly increased, and a substantial number of scientific papers were published studying the role of the striatum in healthy individuals. For the first time, it was possible to assess the contribution of specific areas of the brain during the execution of a cognitive task. Neuroanatomical studies have described functional loops involving the striatum and the prefrontal cortex suggesting a specific interaction between these two structures. This review examines the data up to date and provides strong evidence for a specific contribution of the fronto-striatal regions in different cognitive processes, such as set-shifting, self-initiated responses, rule learning, action-contingency, and planning. Finally, a new two-level functional model involving the prefrontal cortex and the dorsal striatum is proposed suggesting an essential role of the dorsal striatum in selecting between competing potential responses or actions, and in resolving a high level of ambiguity.

## Introduction

The basal ganglia have been a topic of research for more than 100 years. This array of subcortical nuclei located at the base of the brain has intrigued researchers to investigate their contribution to behavior. The putamen and caudate nucleus, nuclei from the basal ganglia collectively referred to as the striatum, have received a lot of interest. Before the early 1990s, the majority of studies were performed on animals following a brain lesion to the area of interest. The main goal of these studies was to assess the specific deficits after carefully producing brain lesions leading to characterization of striatal functions via an animal model. In humans, most studies, that have reported effects of striatal lesions, correlated deficits with the location of the brain lesion either postmortem, or following a traumatic brain injury. However, during that period, investigative methods were not sensitive enough to assess the extent of subcortical injuries with accuracy due to their location in the brain as hidden structures.

With the arrival of modern neuroimaging techniques, such as positron emission tomography (PET) and magnetic resonance imaging (MRI), *in vivo* observation of the striatum became possible and this development opened up the field to study healthy human individuals. Traditionally, relying on data mainly derived from animal research, the striatum was believed to play a role in motor functions only. The striatum was described to be involved in the execution of sequences (Kermadi et al., 1993) and stimulus-response associations (Reading et al., 1991). However, electrophysiological studies have also shown increased anterior striatum activity during the preparation of movements in the “go/no go” task (Kimura, 1992), as well as for coding for specific cues triggering behavior toward a goal (Rolls et al., 1983). In fact, Marsden and Obeso (1994) proposed a dual function of the basal ganglia: the first one would be to foster determined actions to support the creation of new routines, and the second to respond to unexpected circumstances in order to interrupt a sequence of actions to promote a novel action. Not only did they support the idea that the basal ganglia were involved in motor functions, but also in cognition, possibly working in the same manner for both domains. Various studies have shown the involvement of the basal ganglia in a wide range of impairments such as obsessive-compulsive behaviors (Baxter et al., 1987), speech production (D'Esposito and Alexander, 1995), Parkinson's disease (Kish et al., 1988), Tourette's syndrome (Stahl et al., 1982) and others. The proposed role of the basal ganglia in cognition in general was later reiterated by Lieberman who argued for a general contribution of the basal ganglia in cognition in terms of the organization of actions or thoughts (Lieberman, 2000, 2002). He proposed that the basal ganglia were involved in “sequencing” the different aspects of the individual subcomponents in order to produce a coherent behavior. This sequencing of elements is crucial for learning new habits, but also to manipulate these elements in order to adapt to the environment. Hence, the basal ganglia would play a crucial role in language production, but also in a wide variety of behaviors from mastering new motor skills (e.g., walking) to adapting cognitive strategies due to new contingencies (set-shifting). In support of this interpretation, several teams have reported involvement of the basal ganglia, more specifically the striatum, in cognition following clinical observations.

Several models have been proposed in order to explain the role of the striatum in relation to the cortex. Graybiel (1998) has proposed that the basal ganglia were particularly involved in regrouping building blocks of a motor or cognitive behaviors. These buildings blocks were referred to as “chunks,” that can be treated as entities and processed as a unit in which case a habit will be created. Computational models based on reinforcement learning systems provided the underlying mechanisms for this process: (1) the agent would evaluate the action value, (2) the selection of behavior would be accomplished from the assessment of the values of each alternative behaviors, (3) the result associated with the action performed would update the action value for each alternatives (Sutton and Barto, 1998). Indeed, in the context of the striatum, dopaminergic neurons were shown to play a role in encoding the action value (Samejima et al., 2005), which is described as the weight of the different actions regardless of the outcome. Once the action is performed, the output of the chosen action would be encoded as well in terms of its value (Samejima et al., 2005). The striatum has been shown to fire before the execution of a selection, supporting the idea of the evaluation between the potential choices, with the possibility of the subsequent firing serving as a monitoring process to update the previous values for each possible choice.

One can distinguish between two major types of models with respect to the involvement of the prefrontal cortex and the dorsal striatum in cognitive function. The first type stresses the importance of the striatum in learning, such as in implicit learning (Doyon et al., 1997), reinforcement learning (Sutton and Barto, 1998), and “chunking” of actions (Graybiel, 1998). The second type of model stresses the importance of the striatum observed in various cognitive tasks mostly involving goal-directed behavior suggesting a particular role in cognition in general (Marsden and Obeso, 1994; Lieberman, 2002).

The aim of this manuscript is to review findings on the potential role of the striatum in cognition and its interaction with the prefrontal cortex in the context of goal-directed behavior and stimulus-response association involving executive processes, addressing a possible specific common function of the striatum while interacting with the prefrontal cortex. We also discuss these findings regarding the role of the striatum in the context of implicit learning.

First, we describe the anatomical and functional organization of the basal ganglia focusing on the striatum. Then, the relationship between the prefrontal cortex and the dorsal striatum in different functions will be thoroughly characterized. The role of the putamen in cognition and the caudate nucleus is discussed with regards to its contribution in set-shifting, self-initiated actions, rule learning and action-contingency, planning and bilingualism In particular, we review different functional neuroimaging studies that support this notion including those of our own group. Based on these previous studies, we developed a theoretical model involving prefrontal cortex and dorsal striatum interaction and their contribution to cognition. In line with previously proposed theories, we hypothesize that the dorsal striatum plays a rather specific role in resolving ambiguity when there is a high level of competition between possible alternatives (linked to either the similarity or complexity of the stimuli), insisting on the rule or set generation required to choose an action. We also provide evidence that this role is independent of the type of stimuli being processed and may not contradict the role of the striatum in implicit and sequence learning.

## Anatomical description

**Figure 1:**
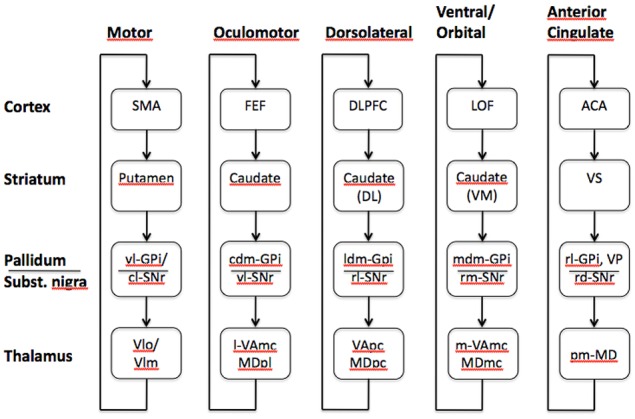
**Functional loops as described by Alexander et al. (1986) involving the prefrontal cortex and the basal ganglia**. Specific areas of the prefrontal cortex will interact with specific nuclei within the basal ganglia generating five closed parallel striato-thalamo-cortical loops, the dorsolateral, and motor loops involve the caudate nucleus and the putamen, respectively. The oculomotor and ventral circuits involve different areas of the caudate nucleus, while the anterior cingulate loop interacts with the ventral striatum. SMA, supplementary motor area; vl-GPi, ventrolateral-globus pallidus internal segment; cl-SNr, caudolateral substantia nigra pars reticulata; VLo, ventrolateral nucleus of the thalamus pars oralis; Vlm, ventrolateral nucleus of the thalamus pars medialis; FEF, frontal eye fields; cdm-GPi, caudodorsomedial globus pallidus internal segment; vl-SNr, ventrolateral substantia nigra pars reticulata; l-VAmc, lateral ventral anterior nucleus of the thalamus pars magnocellularis; MDpl, parvocellular subnucleus of mediodorsal nucleus of the thalamus; ldm-GPi, lateral dorsomedial globus internal segment; rl-SNr, rostrolateral substantia nigra pars reticulata; VApc, parvocellular portion of the ventral anterior thalamic nucleus; MDpc, parvocellular portion of the mediodorsal thalamic nucleus; LOF, lateral orbitofrontal cortex; Caudate (VM), ventromedial caudate nucleus; mdm-GPi, medial dorsomedial globus pallidus internal segment; rm-SNr, rostromedial substantia nigra pars reticulata; m-VAmc, medial ventral anterior nucleus of thalamus magnocellularis; MDmc, magnocellular subnucleus of mediodorsal nucleus of the thalamus; ACA, anterior cingulate area; VS, ventral striatum; rl-GPi, rostrolateral globus pallidus internal segment; rd-SNr, rostrodorsal substantia nigra pars reticulata; pm-MD, posteromedial mediodorsal nucleus of the thalamus (adapted after Alexander et al., 1986).

The anatomy of the basal ganglia has been largely described in the past few years (Nolte, 2002; Haber and Gdowshi, 2004; White, 2009). The basal ganglia consist of an array of subcortical nuclei—including the caudate nucleus, the putamen, the globus pallidus, the subthalamic nucleus, the substantia nigra, and the nucleus accumbens, and are mostly found in the basal telencephalon and diencephalon. In humans and primates, the caudate nucleus and the putamen collectively form the striatum, while the most ventral part of the putamen and caudate nucleus with the nucleus accumbens are referred to as the ventral striatum. In rodents, the internal capsule separating the caudate nucleus from the putamen is absent making it impossible to isolate these structures. For this reason, the caudate nucleus and putamen in rodents are usually referred to as the dorsal striatum. The striatum and subthalamic nucleus constitute the input structures of the basal ganglia, while the substantia nigra and the internal segment of the globus pallidus (GPi) are usually identified as the output nuclei. Basal ganglia have been shown to receive topographically organized projections from various regions of the cerebral cortex (Delong and Wichmann, 2007), including the temporal lobe (Middleton and Strick, 1996), parietal lobe (Selemon and Goldman-Rakic, 1988), and the brainstem (Bostan and Strick, 2010). Furthermore, a large amount of afferent projections come from the frontal lobe suggesting an important functional fronto-striatal interaction (Selemon and Goldman-Rakic, 1988; Stanton et al., 1988; Yeterian and Pandya, 1991). These rich interconnections seem to play a key role in various functions associated with the limbic, oculomotor, motor and cognitive systems. Pioneering work by Alexander et al. (1986) describes five functional subcortico-thalamo-cortical loops implicating different regions of the prefrontal cortex (PFC) and striatum (Figure 1). These closed loops were first reported to be parallel, and subsequent neuroanatomical work (Wiesendanger et al., 2004) and neuroimaging studies (Lehéricy et al., 2005; Leh et al., 2007) supported this idea. However, even if the information seemed to be segregated in these loops, studies in primates have shown some convergence and overlapping at the level of the basal ganglia suggesting possible integration, specifically in the striatum (Flaherty and Graybiel, 1991, 1994). These findings are still a matter of debate that goes beyond the scope of this manuscript.

With respect to the general organization, the striatum and subthalamic nucleus receive excitatory glutamatergic inputs from the cerebral cortex. The striatum projects to the GPi via monosynaptic inhibitory gamma-aminobutyric acid (γ-GABA) connections. After receiving the signal the GPi sends inhibitory γ-GABAergic projections to the thalamus, which then transfers excitatory projections to the cerebral cortex closing the loop. This is referred to as the “direct pathway” which will favor a behavior. Additionally, there is an “indirect pathway” by which the striatum sends inhibitory projections toward the external segment of the globus pallidus (GPe), which in turn will send γ-GABAergic projections to the subthalamic nucleus. The subthalamic nucleus then sends excitatory projections to the GPi, which inhibit the thalamus and reduce cortical excitation. This “indirect pathway” has an overall inhibitory effect on behavior. Lastly, a third “hyperdirect pathway” has been characterized: the cerebral cortex sends excitatory projections directly to the subthalamic nucleus bypassing the striatum and stimulating the GPi by inhibiting the thalamus, leading to movement suppression. It has been proposed that this “hyperdirect pathway” acts as a regulatory mechanism to inhibit unwanted movements.

Evidence supporting the existence of fronto-striatal loops, as previously proposed by Alexander et al. (1986), has been obtained through animal studies (Haber, 2003; Calzavara et al., 2007) and through studies in humans (Lehéricy et al., 2004; Leh et al., 2007). Different studies have shown a contribution of the basal ganglia capable of supporting various behaviors (Divac et al., 1967). In monkeys, it was previously shown that lesions located in the anterior portion of the caudate nucleus caused task switching impairments during working memory tasks (Rosvold et al., 1958) and inhibitory tasks (Battig et al., 1962). In cats, similar types of lesions resulted in impairments in the early stage of learning or retrieval of stimulus-response associations (Prado-Alcala et al., 1973; Prado-Alcalá and Cobos-Zapiaín, 1979). Furthermore, caudate lesions were shown to introduce behavior of perseverance in the face of two potential responses (Olmstead and Villablanca, 1979). In rodents, several studies emphasized the involvement of the basal ganglia in memory systems, such as stimulus association learning and procedural learning (Packard et al., 1989; Mcdonald and White, 1994).

In humans, several studies have reported cases in which alterations of the striatum led to prefrontal cortex dysfunction. This is the case in Parkinson's disease, a neurodegenerative disease characterized by alteration of the dopaminergic neurons in the substantia nigra pars compacta (Albin et al., 1989) affecting the dorsal striatum gradually and then moving toward the ventral striatum (Kish et al., 1988). Clinical observations have reported greater deficit in executive processes including set-shifting and planning (Taylor et al., 1986; Owen et al., 1992; Taylor and Saint-Cyr, 1995; Dagher et al., 2001). For instance, Parkinson's patients in the off-medication state display strong impairment in the rule-switching paradigm due to their incapacity to apply a different rule either from perseveration or learned irrelevance (Owen et al., 1993). Their attentional flexibility is reduced and the ability to switch between two tasks becomes impaired (Cools et al., 2003). In some cases of Parkinson's disease, specific impairments have also been observed in syntax comprehension (Hochstadt et al., 2006) and speech production (Lieberman et al., 1992). Other clinical cases involving caudate lesions caused by brain trauma have reported specific impairments in executive functions, strengthening the hypothesis of fronto-striatal interactions in behavior (Richfield et al., 1987; Mendez et al., 1989; Degos et al., 1993; Pickett et al., 1998), even if some studies have suggested a greater role in visuo-spatial processing (Karnath et al., 2002). The deficits observed following striatal lesion or dysfunction are strikingly similar to frontal lobe patients (Owen et al., 1990) suggesting furthermore, a strong functional bond between the striatum and the prefrontal cortex. In summary, these previously reported studies point toward an involvement of the striatum in working memory and cognitive flexibility, both processes usually associated with frontal lobe functioning. Moreover, it has been proposed that specific alterations of any of the five fronto-striatal loops, previously described by Alexander et al. (1986), could lead to specific behavioral deficits ultimately resulting in well-defined syndromes (Cummings, 1993).

## Fronto-striatal relationship

In humans, various observations were made linking striatal and prefrontal activity. The prefrontal cortex is known to play an important role in executive functions, which refer to mental processes that enable an individual to plan and organize personal strategies toward goal-directed actions (Baddeley, 1986). These strategies include planning, inhibition, selective attention, mental flexibility as well as manipulation of information within working memory. Extended lesions in the prefrontal cortex can cause significant impairments in all of these processes (Milner, 1982). Functional MRI studies have revealed co-activation of the striatum and the prefrontal cortex during performances associated with executive functioning (Cools et al., 2002; Hedden and Gabrieli, 2010). Furthermore, using PET imaging, striatal dopamine release has been associated with working memory capacity in healthy volunteers (Cools et al., 2008; Landau et al., 2009). Finally, studies of Hampson et al. (2006) and others have reported increased functional connectivity between prefrontal cortex and striatum during the performance of working memory tasks. Thus, increasing evidence points to specific contribution of the striatum in executive functions.

### Putamen: Purely motor functions?

It has been proposed that the putamen might contribute specifically to motor processing while the caudate nucleus might be involved in the cognitive aspects of behavior. In primates, it has been described that the putaminal region received large amounts of projections from various motor regions of the cortex, including the primary motor cortex, premotor cortex, supplementary motor area (SMA), cingulate motor areas, and also from the motor nuclei of the thalamus (Mcfarland and Haber, 2000). Convergence of these inputs suggests a possible integration and/or modulation of motor information at the level of the putamen. Similar results were observed using diffusion tensor imaging, by which tract reconstruction highlighted pathways between the posterior portion of the putamen and the premotor, motor and SMA regions (Lehéricy et al., 2004). Furthermore, the anterior putamen revealed tracts heading toward the pre-SMA and the lateral premotor cortex suggesting again well-defined segregated pathways while supporting a particular contribution of the putamen in motor functions. Numerous functional studies have addressed the possible association of the putamen with motor functions. More accurately, it has been proposed that the putamen is contributing to movement preparation during self-initiated behavior (Alexander, 1987). A PET study by Jenkins et al. (2000) has revealed significantly greater activation of the putamen and the anterior SMA during a paced self-initiated compared with a cue-initiated finger tapping task. Replication of these results were obtained in a study by Cunnington et al. (2002) using fMRI. Indeed, significantly increased activity of the putamen was reported during the self-initiated finger sequence as opposed to generating the same sequence following an auditory cue. Furthermore, early activity in the pre-SMA prior to the movement suggested a possible involvement in motor preparation. According to some authors, the movement rate associated with a self-initiated motor sequence is mainly linked with activity in the putamen (Taniwaki et al., 2003). It is well-established that the putamen has strong interactions with the motor systems. However, the possibility exists that the putamen could extend its activity beyond the exclusive function of motor processing as it was traditionally thought. Functional connectivity analysis, using the putamen as a seed region, has revealed strong co-activation of the latter with prefrontal regions (Marchand et al., 2008), which could suggest a potential role in cognition. This interaction between the putaminal region of the striatum and the prefrontal cortex has also been observed in primates during the preparation of a movement (Romo et al., 1992; Schultz and Romo, 1992). In humans, prefrontal and putamen activities during motor tasks have been repeatedly observed (Gordon et al., 1998; Jenkins et al., 2000; Cunnington et al., 2002; Elsinger et al., 2006). Some researchers have addressed this issue by looking at motor production and the pattern of cerebral activity of both hands, considering that differential use of the hands would affect proficiency at executing a motor task. In their paper, François-Brosseau et al. (2009) asked healthy young participants to perform a self-initiated and externally-triggered finger task, using one hand at a time, in which a motor sequence was executed by pressing buttons on a response box during an fMRI session. The control condition involved a repeated selection of the same stimulus following a visual cue. Their results revealed a significant increase of putamen involvement during the self-initiated condition when comparing the right hand over the left hand execution. In contrast a greater putaminal activation was observed in the control condition when the left hand was compared to the right hand. The authors interpreted their results by proposing a specific contribution of the putamen to the execution of unfamiliar movement sequences. However, the use of a non-dominant hand may interfere with the proper execution of the task possibly reallocating task demands. Consequently, the control condition, considered a simpler task, may reflect similar demands as the self-initiated condition for the right hand. The authors suggested that the putamen was particularly involved in the execution of novel motor actions. Interestingly, using functional MRI, it was reported that the administration of a D2-receptor agonist in high impulsive individuals, increased putamen activity significantly when switching attention toward a new category and improved performance on the task (Cools et al., 2007).

The involvement of the putamen has also been detected during implicit motor sequence learning (Grafton et al., 1995; Doyon et al., 1996; Jueptner et al., 1997). Interestingly, during the learning phase, greater activity was observed in the anterior associative portion of the putamen, but as learning progressed in time, a shift toward the posterior part of the putamen, usually referred to as the sensorimotor area was observed (Lehéricy et al., 2005). This region interacts more with parietal cortices and is thought to help consolidating a motor sequence. This type of learning is due to incremental exposure of a sequence and seems to interact with the brain differently than a motor adaption task (see Doyon et al., 2009 for extensive review). Categorical classification studies have also reported increased putamen activity during the learning process, but also during simple associations (Degutis and D'Esposito, 2007; Helie et al., 2010). Interestingly, when the features of the target stimulus are less distinguishable within the different categories, the putamen involvement was increased. For example, Degutis and D'Esposito (2007) had subjects perform a categorization task, in which they classified face stimuli based on the width between the eyes and the length of the nose. Faces with longer noses and greater width between the eyes were part of the first category, while faces with a smaller distance between the eyes and shorter noses were associated with the second category. Subjects were initially trained on the task prior to the fMRI session. Functional MRI results showed significantly greater putamen and PFC activity when stimuli were closer to the category boundary. DeGutis and D'Esposito proposed that the striatum as well as the PFC responded to the level of ambiguity associated with stimuli close to the boundaries of the categories.

### Caudate nucleus and cognition

The caudate nucleus has drawn much attention to its possible functional interaction with the prefrontal cortex. As it was previously shown by the identification of fronto-striatal loops (Alexander et al., 1986), the possible interaction between the caudate nucleus and the lateral prefrontal cortex has been addressed in the context of executive functions. In the next few paragraphs, the fronto-striatal interaction via specific processes including set-shifting, planning cognitive self-initiated actions, rule learning, action-contingency, and bilingualism will be discussed.

#### Set-shifting

The involvement of the fronto-striatal region has been reported in set-shifting tasks numerous times in the literature (Rogers et al., 2000; Monchi et al., 2001, 2006b; Lewis et al., 2004). The concept of set-shifting can be defined as the ability to change our attention from one response set to another according to the changing goals of a task. Clinical observations from Parkinson's disease patients have already raised strong concerns about their ability to maintain and/or shift their attention to another mental set (Cools et al., 1984; Flowers and Robertson, 1985; Owen et al., 1993). These impairments were not only observed in complex tasks, but also in simple selection tasks in which a specific rule needed to be applied, highlighting an impairment in cognitive flexibility. Flowers and Robertson showed such a deficit in the context of a simple-shifting task, the Odd-Man-Out, in which three shapes (e.g., a small triangle, small circle, and a big circle) or three letters (e.g., lower-case “g,” a lower-case “d,” and a capital “D”) were presented and the patient needed to select according to one specific rule (i.e., size or shape) and stick with the same rule during the subsequent trials. After 16 trials, the same stimuli were presented again, but the alternative rule was required for the selection. Parkinson's disease patients showed greater deficit in performing the second round of selection suggesting impairment in maintaining a new rule.

Monchi et al. (2001) studied set-shifting with regards to fronto-striatal activation using a modified computerized version of the Wisconsin Card Sorting Task (WCST) in healthy young adults. This task had been previously used as a neuropsychological test to assess cognitive flexibility following frontal damage (Milner, 1963), even though some caveats were raised in this respect (see Nyhus and Barceló, 2009 for a complete review). During this task, four reference cards are presented in the top of the screen on which the following array of stimuli is observed: one red triangle, two green stars, three yellow crosses, and four blue circles. On each trial, a different test card is presented, and the participant is asked to match the test card to one of the four reference cards according to one of the three features (i.e., color, shape, or number). A feedback is provided after each pairing indicating if the matching rule is to be maintained or not. After a certain number of correct pairings, the rule is suddenly changed without the participant's knowledge, and another rule needs to be applied. In one study using fMRI, Monchi et al. (2001) reported a significant increase of activity of the ventrolateral prefrontal cortex (VLPFC) and caudate nucleus during the reception of negative feedback indicating that a shift to a new response set is required, while the putamen was found to be significantly active with the posterior PFC/premotor cortex during matching following a negative feedback suggesting an involvement in the execution of novel actions. Similar results were replicated by Specht et al. (2009) in their variant of the WCST in which they disentangled the brain activity related to the task specifically from working memory. As previously shown, fronto-striatal regions were significantly activated including the lateral PFC and the caudate nucleus. Indeed in the case of the WCST, the fronto-striatal regions seem to depend on the process involved in identifying the proper rule, but more specifically in the switching from one rule to another following an incorrect pairing (Monchi et al., 2001, 2006b; Lie et al., 2006). These data obtained through fMRI studies were later supported by PET which revealed a release of striatal dopamine during the planning and execution of a set-shift (Monchi et al., 2006a). Furthermore, with transcranial magnetic stimulation (TMS), it has been shown that continuous theta burst impulses directed toward the left dorsolateral prefrontal cortex (DLPFC) interfered with dopamine release of the left striatum, but more importantly these stimulations impaired the performance of a set-shifting task (Ko et al., 2008). A question that remained though is whether this role assigned to the dorsal striatum was stimuli specific, domain specific or more general. In order to start addressing this question, Simard et al. (2011) reproduced similar results following a word variant of the WCST. In their task, the four reference cards were replaced by four words, which represented each a specific category (i.e., boat, transportation; spider, animals; clock, objects; pepper, vegetables). On each trial, the test word needed to be matched with one of the four reference words according to its attack, its rhyme, or its semantic. Similar to the WCST, the same rule of association needed to be maintained for the subsequent trials. A significant increase of activation was found in the same fronto-striatal regions, namely the VLPFC and the caudate nucleus, following a negative feedback when planning a set-shift is required Comparing the results from the two different versions of the WCST revealed that the same striatal process was used whether distinguishing between features of objects or lexical properties of words.

#### Caudate nucleus and self-initiated response

Several clinical cases involving striatal lesions have been reported showing set-shifting impairments. For instance, Pickett et al. (1998) have described a 45 years old female with bilateral lesions in the putamen and caudate nucleus. This particular patient showed mild impairment in the WCST. Furthermore, perseveration was also observed during the performance of the “Odd-Man-Out” task. In this task, three geometric shapes are presented on a card (e.g., a small triangle, a large triangle and a small oval), and the patient is required to select the item that does not match the two others, according to its shape or size, and to keep on selecting the proper item according to the chosen attribute. After a series of 10 cards, the patient is required to perform the same task with the previous cards, but by selecting according the other attribute. During the second part of the task, the patient was unable to switch to the other rule to successfully perform the task, suggesting mental flexibility deficits leading to perseverance. The authors proposed that the impairments are the results of an inability to switch from one subtask to another following a proper sequence of action (motor or mental) in order to successfully attain their goal. Set-shifting deficits have also been observed in PD patients performing the “Odd-Man-Out” task; however in these cases, the deficit has been attributed to their impairment in maintaining task sets, rather than perseveration *per se*.

In the context of the studies previously presented, the caudate nucleus clearly interacts with the PFC, especially when a new response set needs to be selected in the context of a shift. However, a question worth asking is, which component of the set-shifting process drives the caudate nucleus activity, and more specifically whether this activity is dependent on other contextual information. Monchi et al. (2006b) executed an fMRI study with a new sorting task, the Montreal Card Sorting Task, in which a cue card is presented and disappears prior to a series of trials. In the retrieval condition, the cue card needs to be compared against the test card to retrieve the shared attribute, and then the proper reference card is selected according to this attribute. A shift in classification occurs when two consecutive test cards shared different attribute with the reference cards, as this attribute is effectively the rule to be used for matching. In another condition, the “continuous shift,” no cue card is presented but new test cards have to be matched that only shared a single attribute with only one of the four reference cards. As such, only one selection is possible. In this continuous shift condition, test cards are presented so that a different rule is requested on each trial, but it is in essence implicitly given by the task unlike shift trials in the retrieval condition. When shifts in the retrieval condition were compared to either the continuous shifts or the control matching, caudate nucleus activity reached significance with the VLPFC, but significant activation was only found in the VLPFC and not in any part of the striatum when continuous shift trials were compared to the control trials. This indicated that the caudate nucleus activity was not so much modulated by the shift *per se*, but by having to make some mental manipulations to initiate a choice (Monchi et al., 2006b). However, two possibilities could have accounted for this in the described experiment: (1) not being given the rule for classification or (2) having more than one exemplar to choose from, both of which occurs in the shift trial with retrieval but not in the continuous shift condition. A study by Provost et al. (2012) addressed this issue in an fMRI study in which the rule was explicitly given before each pairing. Their analysis revealed a significantly increased activity of the caudate nucleus when set-shifts were performed continuously compared to a control condition, which consisted of pairing the test card with its twin reference card. This indicates that caudate nucleus might still be required even if the rule is given. Furthermore, when analyzing the BOLD signal associated with maintaining the same rule for a long period, they showed that the caudate activity peaked at the first trial (i.e., corresponding to the initial set-shift from the previous condition), but that this activity was maintained for a couple of trials following the set-shift after decreasing below significance. These results suggest that set-shifting should be considered as a gradual process, and that the caudate nucleus seems to play a key role in that process. Moreover, the caudate nucleus is involved in set-shifting when potential conflicting choices are present, and its contribution is required until a rule is used continuously for multiple trials.

#### Rule learning and action-contingency

Rule learning and action-contingency learning are often mentioned in the literature of the caudate nucleus. Numerous studies have reported contribution of the caudate nucleus in this context, but more importantly during probabilistic classification (Poldrack et al., 1999; Seger and Cincotta, 2005). Probabilistic learning depends largely on the observed outcome: if the outcome promotes the initial association, then a behavior that led to this outcome will have a greater possibility of being reproduced again. A certain level of uncertainty is inherent to a probabilistic task where an association between a stimulus and an outcome depends on the probability that the desired outcome is obtained. Once again, the caudate nucleus seems to play a crucial role when an automatic choice is not available similarly to the set-shifting context. Hence, one's judgment is influenced by the number of possible stimuli to choose from, but also by the possible number of outcomes. However, once an association persists in time, the level of ambiguity diminishes leading to a more automatic response. Indeed, the involvement of the caudate nucleus is often reported in the early phase of an association learning supporting its role in probabilistic learning (Seger and Cincotta, 2005).

Similar caudate activation can be observed in action-contingency studies, in which the action would have a potential effect on the outcome (Delgado et al., 2000; Knutson et al., 2001; Tricomi et al., 2004). However, significant caudate nucleus activation was also observed in typical rule learning tasks independently of whether a probabilistic component was present at one point or not (Seger and Cincotta, 2006). Indeed, learning arbitrary visuo-motor associations has shown a contribution of the fronto-striatal regions, including the caudate nucleus (Toni and Passingham, 1999).

#### Caudate nucleus and planning

Similarly to what was perceived with the putamen regarding the execution of novel actions, the caudate nucleus can be characterized as key player in cognitive tasks requiring planning of self-initiated actions. A series of studies have shown the involvement of the fronto-striatal circuit during the execution of the Tower of London planning task. In the traditional version of the task, an array of three beads, each a different color, are displayed in a certain configuration on a 3-column stand. Then another configuration of the beads is presented to the participant whose task it is to reconfigure the initial display of beads with the minimum number of moves. There are various levels of difficulty depending on the minimum number of moves required, but that can be broadly defined into two categories: one that requires 2–3 moves (i.e., simple planning) and one that requires 4–5 moves (i.e., difficult planning). In this context, the difficulty resides in the planning of the movements of the beads before the actual execution. Using PET, studies have shown the involvement of fronto-striatal regions, involving the caudate nucleus, during the execution of the difficult planning compared to the simpler planning condition (Owen et al., 1996) and control condition (Baker et al., 1996). Similar results were obtained using fMRI in which again, caudate nucleus and DLPFC were significantly activated in the planning condition (Van Den Heuvel et al., 2003). Furthermore, a positive correlation was established between the increasing task load and the caudate nucleus activity; these results were replicated subsequently (Provost et al., 2010).

An important issue could be raised of whether task load by itself is sufficient to increase the fronto-striatal activity or whether it depends on the type of cognitive manipulation, while the information is maintained in working memory. With a simple experimental design, Lewis et al. (2004) have brought support to this potential role. Using fMRI, participants had to first maintain in memory four letters visually presented. Then a cue word was presented indicating the task required by the participant. Three experimental conditions were used: simple retrieval of the letters in order; simple manipulation in which participants recalled the third and fourth letter followed by the first and second letter; complex manipulation in which participants needed to recall the first, the third, the second and the fourth letter in that order. Their results showed a significant increase of the caudate nucleus during both manipulations conditions as opposed to the retrieval condition and maintenance respectively. Thus, the caudate nucleus seems particularly implicated when manipulation of information within working memory is required as opposed to simple maintenance. The previously described study from our group using the Montreal Card Sorting Task (Monchi et al., 2006b) gave further support to this possibility, as no significant activation of the striatum was observed when comparing the retrieval without shift condition, which involved maintenance of the cue card in working memory, with either the control or the continuous shift condition, which did not. Significant activation occurred together in the caudate nucleus and the DLPFC in the condition that required manipulation of information in working memory in Lewis et al. (2004) study. Monitoring the information maintained in working memory has also shown to modulate caudate nucleus activity. Here, monitoring processes are defined as keeping track of selections or event-occurrence in one's mind while simultaneously updating the stimuli that remains to be selected or to occur. The DLPFC has been proposed to play a key role in this process (Petrides, 1995). Two types of monitoring have been described: (1) the self-ordered monitoring in which the selection process allowing the tracking of events requires self-generated choices, and (2) the externally-triggered monitoring in which these events or selections are performed by an external source, but the tracking is still self-generated. A study by Provost et al. (2010) comparing the activity associated to the two types of monitoring revealed a specific contribution of the caudate nucleus during the self-ordered monitoring which contrasted with externally-triggered monitoring. This result suggested a contribution of the caudate nucleus specifically when the generation of novel responses is required and when the individual initiates the strategy. This interpretation is the cognitive equivalent of the putaminal function proposed earlier.

#### Involvement of the striatum in bilingualism

Another area of research where striatal activation is regularly reported is bilingualism. Some of the first neuroimaging studies have shown distinct patterns of activation when words are being produced in a second language (i.e., L2) as opposed to the native language (i.e., L1; Klein et al., 1994; Price et al., 1999). The network observed during the production of L2 is fairly similar to the L1 network with the exception that larger activations were found in the language-associated regions and the involvement of the striatum was greatly increased (Abutalebi and Green, 2007). These differences depend on the level of proficiency and age of acquisition of the second language (Perani et al., 1998). Once an individual masters L2, these brain regions characterizing L2 seem to disappear. One thing that is fairly important in using a second language is the ability to switch from one frame of reference to another. Both putamen and caudate nucleus activation have been observed and different roles have been proposed for these structures. On one hand, the putamen could play a key role in the articulation process. Indeed, patients with permanent lesions or patients who underwent temporary disruption of the putamen showed persistent dysarthria (D'Esposito and Alexander, 1995; Robles et al., 2005). Increased activation of the putamen was also observed in multilingual individuals during language production, if these individuals were not highly proficient (Abutalebi et al., 2013). On the other hand, the caudate nucleus could monitor for accuracy and cognitive control (Robles et al., 2005), as well as perseverance (Caplan et al., 1990; Kreisler et al., 2000). It was suggested that the caudate nucleus could be involved in the resolution of ambiguity specific to language processing (Ketteler et al., 2008). This interpretation of the role of the caudate nucleus converges with the work done on the set-shifting process in our group (Monchi et al., 2001; Provost et al., 2012). Stocco and Prat (2014) have proposed that bilingualism is supported by greater executive functioning allowing rapid switching from one language to another, and that the executive advantage might be transferable to greater global cognitive flexibility and better adaptation to executing new rules. In their study, a group of bilingual individuals was compared to a group of monolingual individuals on a revised rapid instructed task learning, in which new instructions were given before each task. The results highlighted faster reaction times for the bilingual group and a greater modulation of the striatum for the bilingual individuals. The contribution of the striatum in language could indeed reflect a more global mechanism than being specific to language *per se*, although some results showed the opposite (Wang et al., 2012).

## Discussion and proposed fronto-striatal functional model

As discussed above, studies performed in the last two decades, have accumulated a substantial amount of data supporting the “action value” model suggesting a strong contribution of the striatum in stimulus-response associations (Hassani et al., 2001; Haruno et al., 2004; Tricomi et al., 2004; Pasquereau et al., 2007; Lau and Glimcher, 2008; Kimchi and Laubach, 2009). Furthermore, it was proposed that part of the striatum could be involved in the encoding of reward prediction errors (Apicella et al., 2009; Kim et al., 2009), possibly to facilitate subsequent updating of information (Stalnaker et al., 2012). However, in the context of goal-directed behavior, especially in humans, one could imagine that a decision is made based on a number of different information influencing the motivation to choose one alternative over the other. Indeed, when learning is acquired to the point of becoming a habit, Balleine and Dickinson (1998) suggested that at least three different stages would occur: Firstly, a contingency learning promotes an association between the action and an outcome. Then an incentive learning occurs in order to assign an appropriate weight to the reward, which could be subject to devaluation after exposure. Thirdly, after multiple expositions to the same pairing, a stimulus-response association predominates the decision making process regardless of the incentive. In this model each type of learning relies on different parts of the striatum. The study by Provost et al. (2012) looking at the involvement of the dorsal striatum in various trials after a set-shift is consistent with this notion, as it argues for dorsal striatum involvement until a rule is properly acquired. In the contingency learning, which we could refer to as goal-directed learning, evidence showed that the dorsal striatum was responsible of that function by contributing to various evaluation processes leading to the chosen outcome. The striatum has been shown to be involved only when possible alternatives are present, and its contribution ceases when competition is resolved. Overall, these results argue that the role of the striatum in implicit learning including sequence learning, as well as its implication in stimulus-response processes involving executive processes, are not necessarily contradictory.

**Figure 2:**
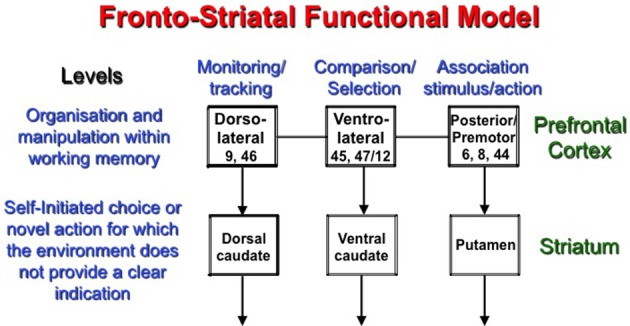
**The model proposes a two-level fronto-striatal organization in which the prefrontal cortex sorts out the information within working memory according to the task at hand and the region responsible for processing, while the proper region of the striatum manages the competition between stimuli (or actions) when novelty and/or indecision is present**. The dorsal caudate nucleus interacts with the DLPFC to support monitoring, while the ventral caudate supports complex comparisons and selections executed by the VLPFC. Finally, the posterior prefrontal cortex is supported by the putamen during self-initiated execution of an action.

As pointed out by Lieberman (2002), the dorsal striatum seems to play a general role in cognition and is seldom involved in ordering adequately various components to create the desired behavior. This central role played by the basal ganglia is applicable not only to executive processes, but also to functions such as language in which a sequence of small actions needs to be perfectly executed in order to express ourselves. These sequences of actions need to be carefully monitored, but more importantly relied on the goal-directed content of the intended speech. From the work that has been presented so far, it is apparent that fronto-striatal regions play an important role in executive processes. The different regions of the striatum seem to interact with precise prefrontal regions in order to assist with specific executive processes when greater planning demands are required or when competition between potential stimuli could interfere with producing an adequate response. The most essential feature of the involvement of the striatum can be detected when the notion of novelty is present. This aspect of novelty is considered during all self-initiated actions that require some sort of processing of information within working memory. In all studies reported above, the involvement of the striatum was observed in task requiring active selection, or updating the state of information within working memory. It was proposed that these processes depend on specific regions of the PFC. Taking the reviewed data into account, we propose a new functional model of the organization of novel information processing within working memory implicating the fronto-striatal pathway (Figure 2). This model has been developed according to the process-specific model of the prefrontal cortex of Petrides (2005) proposing that specific processes are supported by well-defined PFC areas. While different models of prefrontal functional organizations have been proposed, such as the domain-specific model (Goldman-Rakic, 1995; Levy and Goldman-Rakic, 1999), the adaptive coding model (Duncan and Owen, 2000; Duncan, 2001), the hierarchical model (Badre, 2008), or the process-specific model (Petrides, 1995), we favor the Petrides' model. It should be noted that none of these models fundamentally change our proposition for dorsal striatum functioning since, regardless of the specific function associated with the PFC, a highly defined role for the dorsal striatum is assumed. The specific role for the dorsal striatum is characterized as an inherent role similar for all the other brain areas interacting with it.

### Proposed functional model

We propose that the PFC and striatal regions interact in order to plan and/or to execute new actions. In both cases, the idea of self-initiated involvement is predominant. The first level concerns the organization of information in working memory depending on the task at hand. The mid-DLPFC, which occupies area 9/46 and 46, was proposed to play a key role in the monitoring process, which consists of keeping track of the occurrences and non-occurrences of events within working memory. On the other hand, area 47/12 of the VLPFC contributes to the active retrieval process, which allows active comparison between different stimuli held within working memory, and promotes active selection based on specified characteristics. Finally, the posterior region of the PFC is more involved in conditional association between a stimulus and a response. The second level of the model involving the striatum only contributes if a novel action, which involves uncertainty and/or complex manipulation of information, is required. The connection from the DLPFC to the caudate nucleus is more specifically involved in the planning of action, while the VLPFC and the caudate nucleus is more likely linked to its selection, whereas the posterior PFC and the closely related premotor cortex together with the putamen are involved in its execution (Figure 2).

This model was largely inspired by results obtained in our laboratory, as we have shown significant involvement of the caudate nucleus and the DLPFC in self-initiated monitoring (Provost et al., 2010), involvement of the VLPFC and caudate in selecting a new rule (Monchi et al., 2001, 2006b), and activation of the posterior PFC/premotor cortex and the putamen in applying the new rule for the first time or self-initiating different finger movements (François-Brosseau et al., 2009). In addition, other groups have shown the involvement of the putamen and posterior regions of the PFC during the execution of non-routine actions (Cunnington et al., 2002). Finally, we showed a specific contribution of the caudate nucleus during self-initiated monitoring (Provost et al., 2010) and the general process supported by the DLPFC. We propose that the caudate nucleus is solicited when there is a high level of ambiguity affecting distinction between the stimuli. Furthermore, it also applies in higher order functioning, like rule selection. We propose that the level of competition between rules activates the caudate nucleus in order to guide goal-directed behaviors. This theoretical framework insists on the novelty/uncertainty aspect driven by the stimuli. The level of ambiguity increases complexity in this model, sometimes due to the stimuli themselves or caused by the competition between potential responses. We also conjecture that this process is independent of the domain of cognition or the stimuli being processed. This proposed interpretation goes one step beyond other models, and highlights the contribution of the caudate nucleus in a specific process.

Another possible interpretation that needs to be discussed is the possibility that the caudate nucleus might be driven by task difficulty. Indeed, the two concepts are hard to differentiate in the context of executive functioning, especially considering that greater ambiguity can be driven by increasing the number of possible choices. Task difficulty is indeed inherent to more complex cognitive processing, which is relative to the level of novelty and ambiguity. However, it depends on how “task difficulty” is defined and measured. We cannot rule out the effect of task difficulty as being part of the level of ambiguity between stimuli. In consideration of that fact, we argue in favor of the novelty aspect of a task and the ambiguity related to it requiring more cognitive control resulting in an increase of the task difficulty in some instances. As the task difficulty increases, more time and brain resources are usually required to execute a task (Just et al., 1996). In some of the studies reported above, increased striatal activation is not consistent with increased reaction time (Provost et al., 2010). Furthermore, some studies are designed in such a way that specific events of tasks are of interest and cannot necessarily be associated with a reaction time (e.g., negative feedback processing vs. positive feedback processing in the WCST). Yet, the brain resources are indeed different from one condition to another at least with respect to striatal activity. Several studies have investigated the effect of task difficulty without reporting any striatal activation (Grady et al., 1996; Jonides et al., 1997; Gur et al., 2000). However, the involvement of the striatum seems particularly important when the action to be chosen is hard to determine.

## Conclusion

The goal of this review was to investigate the relationship between the dorsal striatum and the prefrontal cortex with respect to executive processes by exposing a functional model. As thoroughly discussed earlier, the dorsal striatum participates in various tasks involving specific processes linked to executive functioning. Our review highlights the possibility that the functional role of the dorsal striatum may be similar across seemingly different processes, such as planning, set-shifting, bilingualism, and finger movements. In all cases, competition between two or more elements is implied, and only a self-generated action from the individual could resolve the conflicting situation. The notion of competition between stimuli and the involvement of the individual into sorting out the possibilities to execute the proper goal-directed behavior seems crucial for the functional contribution of the striatum. Implicitly associated with the planning of an action, the notion of novelty is provided as suggested with the proposed model of fronto-striatal interaction. This proposed model underlines the essential contribution of the striatum in specific situations involving prefrontal activity. As this model was derived from observations in healthy individuals, it would be interesting to examine pathological situations like Huntington's disease or Parkinson's disease. Indeed, neuroimaging data from Parkinson's disease patients showed impairments in set-shifting and self-initiated actions (Monchi et al., 2004; Jubault et al., 2009) supporting the general concept of our model. In finishing, we propose that our model does not necessarily contradict other models stressing the importance of the striatum in implicit learning (Doyon et al., 1997).

### Conflict of interest statement

The authors declare that the research was conducted in the absence of any commercial or financial relationships that could be construed as a potential conflict of interest.
